# Phase‐Change‐Memory Process at the Limit: A Proposal for Utilizing Monolayer Sb_2_Te_3_

**DOI:** 10.1002/advs.202004185

**Published:** 2021-05-14

**Authors:** Xue‐Peng Wang, Xian‐Bin Li, Nian‐Ke Chen, Bin Chen, Feng Rao, Shengbai Zhang

**Affiliations:** ^1^ State Key Laboratory of Integrated Optoelectronics College of Electronic Science and Engineering Jilin University Changchun 130012 China; ^2^ College of Materials Science and Engineering Shenzhen University Shenzhen 518060 China; ^3^ Department of Physics, Applied Physics, and Astronomy Rensselaer Polytechnic Institute Troy NY 12180 USA

**Keywords:** 2D limit, first‐principles molecular dynamics, high‐density data storage, phase change memory, Sb_2_Te_3_

## Abstract

One central task of developing nonvolatile phase change memory (PCM) is to improve its scalability for high‐density data integration. In this work, by first‐principles molecular dynamics, to date the thinnest PCM material possible (0.8 nm), namely, a monolayer Sb_2_Te_3_, is proposed. Importantly, its SET (crystallization) process is a fast one‐step transition from amorphous to hexagonal phase without the usual intermediate cubic phase. An increased spatial localization of electrons due to geometrical confinement is found to be beneficial for keeping the data nonvolatile in the amorphous phase at the 2D limit. The substrate and superstrate can be utilized to control the phase change behavior: e.g., with passivated SiO_2_ (001) surfaces or hexagonal Boron Nitride, the monolayer Sb_2_Te_3_ can reach SET recrystallization in 0.54 ns or even as fast as 0.12 ns, but with unpassivated SiO_2_ (001), this would not be possible. Besides, working with small volume PCM materials is also a natural way to lower power consumption. Therefore, the proposed PCM working process at the 2D limit will be an important potential strategy of scaling the current PCM materials for ultrahigh‐density data storage.

## Graphical abstract

By first‐principles molecular dynamics, this work proposes a phase change memory (PCM) process at the 2D limit that is achieved in a monolayer Sb_2_Te_3_ for the first time. This will be a potential strategy for improving the performance of PCM technology to realize high integrated density, fast speed, low power consumption, or even artificial intelligence applications.

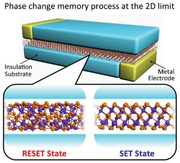

## 1 Introduction

Phase change memory (PCM) is a leading candidate in the emerging nonvolatile memory technology.^[^ ^1^, ^2^, ^3^, ^4^ ^]^ A PCM material, such as the flagship Ge_2_Sb_2_Te_5_ (GST),^[^ ^5^ ^]^ can reversibly and rapidly switch between their amorphous and crystalline phases by a laser or electric pulse, while the two phases can still retain a large contrast both in resistivity and reflectivity.^[^ ^6^, ^7^, ^8^, ^9^ ^]^ PCM materials are also on their way to achieve universal memory,^[^ ^10^, ^11^ ^]^ in‐memory computation,^[^ ^12^, ^13^ ^]^ and for the emerging artificial intelligence (AI) applications.^[^ ^14^, ^15^, ^16^ ^]^ To be used for high‐density integration, scalability is one of the most important criteria that has to be considered. Currently, there are several ways to scale the devices down by developing nanostructures. For example, nanoparticles (0D),^[^ ^17^ ^]^ nanowires (1D),^[^ ^18^, ^19^ ^]^ and thin films^[^ ^20^, ^21^ ^]^ or superlattices^[^ ^22^, ^23^, ^24^ ^]^ or heterostructures^[^ ^25^ ^]^ (2D) have been experimentally demonstrated. When a PCM device is scaled down to the tens‐of‐nanometer scale, the encoding current can also be significantly reduced,^[^ ^18^, ^26^ ^]^ which can be important for lowering the power consumption of PCM devices.

However, as the size going down, a number of issues, which could significantly impact the phase change behavior of PCM materials, arise. For example, when the diameter of GST nanoparticles approaching 17 nm, surface‐induced heterogeneous nucleation becomes dominant, instead of the homogenous nucleation of a bulk material.^[^ ^17^ ^]^ Also, for a thin GST film, less vacancies will form due to substrate stress, which makes the recrystallization difficult. As a matter of fact, when the film thickness is less than 2 nm, the amorphous state can no longer be switched back to the crystalline state.^[^ ^21^, ^27^ ^]^ This implies that the PCM‐based materials, such as the popular GST, will encounter a bottleneck for scaling, especially at the several‐nanometer size scale. Thus, for further scaling, which has become one of the most important challenges in this field, a different strategy is urgently needed.

In this work, by first‐principles molecular dynamics (MD) simulations, we propose that a PCM process may be realized in one‐monolayer Sb_2_Te_3_ (ST), the thinnest so far with only 0.8 nm thickness. At this limit, however, the physical processes are qualitatively different from those in their thicker and bulk counterparts: first, the SET process (recrystallization) happens as a direct amorphous‐to‐hexagonal transition, instead of the usual amorphous‐to‐cubic‐and‐then‐to‐hexagonal transition in bulk.^[^ ^28^ ^]^ Second, the RESET (amorphous) state accommodates electrons with more spatial localization, due to geometrical confinement, which may in fact stabilize the amorphous phase over the crystalline phase. This is good for nonvolatile data storage, as it overcomes the known poor stability of bulk amorphous ST. Third, the interaction between monolayer ST and substrate/superstrate may be used to control the phase change behavior: if sandwiched between passivated SiO_2_ (001) surfaces, the SET time is within 540 ps; if sandwiched by 2D hexagonal Boron Nitride (BN) instead, it can be considerably shorter, only 120 ps. Our work lays the ground for high‐performance PCM applications using thinnest PCM materials possible.

## 2 Results and Discussions

The flagship Ge–Sb–Te alloys for PCM applications may be regarded as a pseudo binary compound between GeTe and Sb_2_Te_3_.^[^ ^29^ ^]^ Usually, ST, without the Ge, is not applicable to PCM applications, because its amorphous state is easily recrystallized even at a relatively low temperature.^[^ ^30^ ^]^ At the nanometer scale, on the other hand, GST becomes difficult to recrystallize due to geometrical constraint.^[^ ^21^, ^27^ ^]^ The disadvantage of ST, being overly easy to recrystallize, could therefore be a potential advantage when the size is significantly reduced.

**Figure 1:**
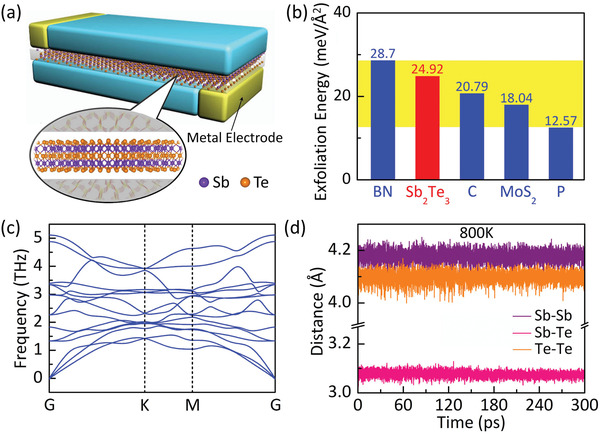
Stability of monolayer ST for PCM. a) Schematic diagram of a 2D PCM cell. Color coding: purple for Sb and orange for Te atoms. b) Exfoliation energy (EE) of monolayer hexagonal BN, Sb_2_Te_3_, graphene [C], MoS_2_, and black phosphorus [P] from their corresponding bulk. c) Phonon spectrum of monolayer Sb_2_Te_3_. d) Average nearest‐neighbor distances between various element pairs: Sb–Sb (purple), Sb–Te (pink), and Te–Te (orange) in monolayer Sb_2_Te_3_ during a 300 ps MD at 800 K.

The ST has a distinct bulk structure from the rest of the GST family, where quintuple‐atomic layers form a compact structure separated from other quintuple layers by weak van der Waals (vdW) interactions.^[^ ^31^ ^]^ This is similar to other layered materials such as MoS_2_ and black phosphorus.^[^ ^32^, ^33^ ^]^ An ST quintuple layer consists of a stack of atoms in the Te–Sb–Te–Sb–Te order with Te atoms terminating both outermost layers. As such, we expect an exfoliation of bulk ST, or growth, can lead to a single quintuple layer or monolayer ST, as depicted in **Figure** **1**a. We have examined the exfoliation energy (EE) of monolayer ST from its bulk form. Figure 1b shows that EE of ST (24.92 meV Å^−2^) is higher than those of graphene (20.79 meV Å^−2^), MoS_2_ (18.04 meV Å^−2^), and black phosphorus (12.57 meV Å^−2^), but smaller than that of hexagonal BN (28.7 meV Å^−2^), even which is considered small. Next, we examine the dynamic stability of monolayer ST. Figure 1c shows the ground state phonon spectrum. There is no imaginary phonon frequency throughout the Brillouin zone. To look into this matter further, we perform a first‐principles MD at 800 K for 300 ps. To reduce the work load, however, the simulation here was done for a freestanding monolayer ST, which is expected to be less stable than the one vdW‐sandwiched between substrate and superstrate. Figure 1d shows the time evolution of the nearest neighbor distances for Sb–Te, Sb–Sb, and Te–Te pairs, which are basically unchanged. This is an indication that the crystal structure of monolayer ST is intact at such a temperature. Hence, the monolayer crystalline PCM is both energetically and dynamically stable, suggesting the feasibility of experimental synthesis or growth.

**Figure 2:**
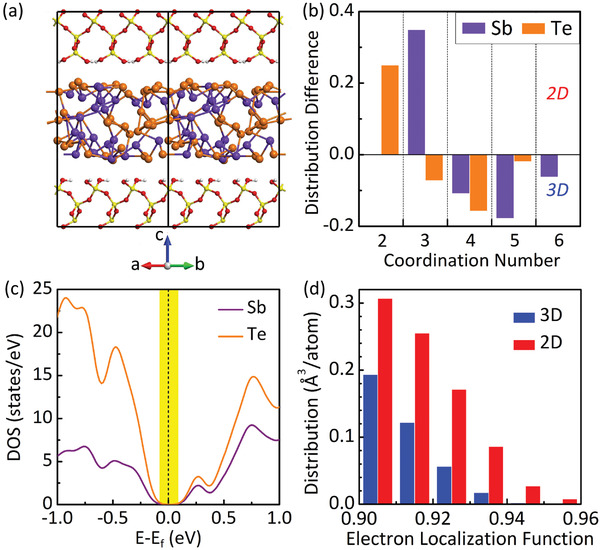
Atomic structure and electronic properties of monolayer amorphous ST. a) Atomic structure of a 2D amorphous ST sandwiched inside SiO_2_ (001) with passivated surfaces. Color coding of Sb and Te is the same as in Figure 1. For SiO_2_, red is for O, yellow is for Si, and white is for H. b) Differences in the distribution of coordination numbers between 2D and 3D amorphous ST. c) DOS of Sb and Te in 2D amorphous ST. Bandgap is highlighted by the vertical yellow bar. d) Volume occupied by electrons versus their localization function in 2D and 3D amorphous ST, respectively.

Our device concept is a monolayer ST sandwiched between two insulating surfaces, as shown in Figure 1a where passivated SiO_2_ (100) surfaces^[^ ^34^ ^]^ have been used. To prevent direct tunneling during operation at this ultrathin limit, the bottom and top electrodes for the device should be horizontally offset,^[^ ^35^ ^]^ as shown in Figure 1a. We obtain the 2D amorphous ST by a melt‐quench RESET MD (see details in the “Experimental Section”). **Figure** **2**a shows its atomic structure. Due to the full passivation of the SiO_2_ surfaces, no interfacial chemical (ionic or covalent) bonds have formed during the melt‐quench simulation. In other words, it retains the structural and electronic properties of a pristine (freestanding) 2D amorphous ST. As a comparison, we also simulated 3D bulk amorphous ST, using the same melt‐quench method. Figure 2b shows the distribution difference in the coordination numbers (CNs) between 2D and 3D (the absolute distributions of the CNs are given in Figure S2 in the Supporting Information). While there are absolute distributions for every CNs in the 2D amorphous ST, the difference increases for CN = 2 and 3 but decreases for CN = 4, 5, and 6 compared with the 3D case, which is indicative of a significant decrease in the total coordination number per atom, CN = Σ*_i_* (CN)*_i_* in the 2D limit.

Based on the calculated density of states (DOS) in Figure 2c, a bandgap exists in the 2D amorphous ST. To understand it, we show in Figure 2d the distribution of the electron localization function (ELF).^[^ ^36^ ^]^ Calculation details of the distributed volume of electrons at a given ELF range can be found in Figure S3 in the Supporting Information. A value >0.9 here is indicative of localized electronic states. When comparing the results between 2D and 3D, it becomes clear that the 2D amorphous ST exhibits a larger degree of localization than its 3D counterpart. This is understood given the large geometrical confinement in the direction normal to the 2D film. Figure S4 in the Supporting Information shows that these atomically localized states mainly consist of Te lone‐pairs, instead of the usual partially occupied dangling‐bond states. The doubly occupied lone‐pair states explain the existence of the bandgap in Figure 2c.^[^ ^37^ ^]^ Lone‐pair states are low in energy; their abundance should help to stabilize the 2D amorphous ST. Indeed, a 1.2 ns (long time) first‐principles MD at 300 K (see Figure S5 in the Supporting Information) confirms the stability of the film.

**Figure 3:**
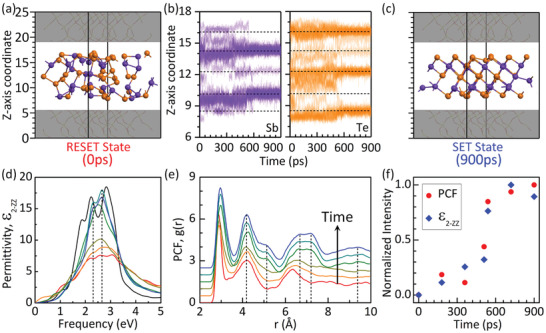
Crystallization process of 2D amorphous ST. Atomic structures of a) initial RESET state and c) final SET state. Color coding of the atoms is the same as in Figure 2. b) Time evolution of the *z*‐coordinates of Sb (purple) and Te (orange) atoms during recrystallization. Five dashed‐lines indicate the standard positions of quintuple layer of crystalline ST. d) Imaginary part of permittivity, *ε*_2‐_ *_ZZ_* and e) PCF at different times of recrystallization: 0 ps (red), 180 ps (orange), 360 ps (dark yellow), 540 ps (green), 720 ps (cyan), and 900 ps (blue). Black line in (d) stands for *ε*_2‐_ *_ZZ_* of a perfect monolayer ST crystal. f) Normalized average peak intensity of *ε*_2‐_ *_ZZ_* and PCF, marked by vertical dashed lines in panels (d) and (e), as a function of time.

For a typical PCM material at nanoscale, the difficulty in recrystallization from the amorphous phase (i.e., the SET process) poses a challenge. Therefore, it can be critically important to understand the recrystallization process and the physical properties of the subsequent 2D material. After a 900 ps annealing at 600 K, the initial amorphous phase (i.e., the RESET state in **Figure** **3**a) transforms into a clearly defined ordered phase (i.e., the SET state in Figure 3c). To monitor this structural change, we plot in Figure 3b the *z*‐coordinate of the atoms during the recrystallization. At *t* = 0 ps, a significant intermixing between the Te layer and adjacent Sb layers can be clearly seen. We should note that the actual degree of disorder can be larger than what is reflected in Figure 3b, because the *z*‐coordinate may not capture disorders in the *x*–*y* plane, which are in fact significant, as can been seen in the 3D plot in Figure 3a.

As the simulation proceeds (*t* > 0 ps), the distribution of the *z*‐coordinates fluctuates without a clear trend and significantly deviates from the standard positions of crystalline ST, which is indicated by the dashed lines in Figure 3b. However, at *t* ≈ 540 ps, a sudden change takes place, which can be visually seen. This change signals a first‐order phase transition to the quintuple‐layered structure of the ordered 2D phase. This phase transition can be confirmed by the energy evolution shown in Figure S6a in the Supporting Information. To examine this transition, Figure 3e shows the pair correlation functions (PCFs) at different times. Peaks and valleys start to develop after *t* = 540 ps with *r* > 5 Å, which signals long range order. In fact, we also propose a 2D PCF to describe the structural order in the *x*–*y* plane. See details for the 2D PCF in Section 6 in the Supporting Information. The 2D PCF also demonstrates a phase transition at 540 ps. For memory applications, a clear optical and/or electrical contrast is necessary. Figure 3d shows the imaginary part of permittivity in the *z*‐direction (i.e., *ε*_2‐_ *_ZZ_*) versus time. Changes in optical signals (cf. ref. ^[38^ ^]^) can be clearly seen: before *t* = 360 ps, peaks in the energy range of 2–3.5 eV increase slowly. Between 360 and 540 ps, however, they increase rapidly. At 900 ps, *ε*_2‐_ *_ZZ_* is already close to that of 2D crystalline ST. The contrast in optical signals suggests 2D ST may be also used to achieve ultrathin optoelectronic applications like Sb thin films.^[^ ^39^ ^]^ There is a strong correlation between the structural property PCF and optical property *ε*_2‐_ *_ZZ_*, as can been seen in Figure 3f, where normalized average peak intensities for PCF and *ε*_2‐_ *_ZZ_*, are shown as functions of time. While such a correlation is essential for reversible phase change applications, this is the first time that it has been demonstrated for 2D ST.

**Figure 4:**
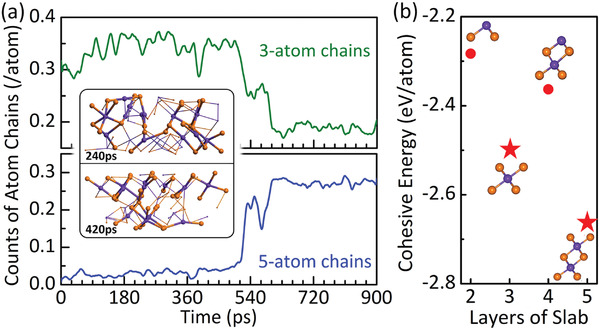
Time evolution of 3‐atom (3‐AC) and 5‐atom (5‐AC) chains during the recrystallization. a) Counts of 3‐AC and 5‐AC versus time. Insets are snapshots at 240 and 420 ps, respectively, in which 3‐AC is highlighted by the large balls and thick sticks. Color coding is the same as in Figure 2. b) Cohesive energy of *n*‐layer Sb–Te crystalline slabs (2 ≤ *n* ≤ 5). The more energetic favored 3‐layer and 5‐layer slabs are highlighted by red stars. Note that *n* layer here corresponds to *n*‐AC chains in the amorphous phase in panel (a).

To examine the recrystallization at atomic level, we first point out that small segments of atom chains (ACs) are characteristic (or fingerprint) of PCM materials.^[^ ^40^ ^]^ These chains must have the correct chemical connectivity, and in the case of crystalline phase they should also have the 180° bond angle, as a result of the p‐bonding of their valence electrons. At an early time of our MD (*t* < 510 ps), 3‐atom chains (3‐ACs) dominate the amorphous phase, as revealed by the count plot in **Figure** **4**a. One may wonder why the 3‐AC is a common feature here. To search for an answer, we calculate the cohesive energy for the crystalline phase, as a function of thickness. The results are shown in Figure 4b. It is found that, besides the quintuple‐layer slab, which is the ground state of the crystalline phase, the 3‐layer Te–Sb–Te slab is the next most stable form of 2D ST. Hence, the dominance of 3‐ACs here in the amorphous phase is a reminiscence of their stability in the crystalline phase.

At *t* ≈ 510 ps, the amount of 3‐ACs starts to decrease precipitately, giving to the formation of (longer) 5‐atom chains (5‐ACs). A dominance of the 5‐ACs is a sign that the crystalline phase has formed. In addition, Figure S6b in the Supporting Information shows the evolution of fourfold ABAB atom rings, which has been demonstrated to play an important role in rapid crystallization of PCM materials.^[^ ^41^, ^42^, ^43^, ^44^, ^45^, ^46^ ^]^ The count of ABAB atom rings also exhibits a significant increase at 510–540 ps. We should note that in the usual (cubic) bulk crystalline phase, the ACs are often noticeably longer. Here, however, we have instead the hexagonal crystalline phase in which after every 5 atomic layers, the chain reaches a vdW gap (=an ordered layer of Sb vacancies). As the presence of the vacancies breaks the 180° chains, it accounts for the shorter ACs in 2D amorphous ST. Importantly, the shorter the chains, the easier the recrystallization.

Figure 4 also reveals how the distribution of 3‐ACs evolves with time: in the early (amorphous) stage, the chain orientations are random, as can be seen from the structural plot at 240 ps in the top inset of Figure 4a. At a later time, say *t* = 420 ps, although the 2D ST is still amorphous, these 3‐ACs have reoriented themselves and more importantly started to order, as judged from both the PCF and permittivity, as can be seen in the lower inset of Figure 4a (also in Figure S7 in the Supporting Information). When this happens, other atoms not part of the chains, but in the vicinity, can readily gather and assemble onto them. In other words, these reoriented 3‐ACs now serve as the template for a quick recrystallization, which becomes pronounced after *t* = 540 ps. Here, we stress that the 2D amorphous ST recrystallizes to the hexagonal phase directly without going through the (intermediate) cubic phase. One may view the hexagonal ST as TeSbTeSbTe□, where □ stands for a Sb vacancy. As a matter of fact, the so‐called vdW gaps of ST are nothing but the ordered Sb vacancies. In other phases, crystalline cubic or amorphous, such vacancies always exist, as they are an important component to fulfill the chemical composition Sb_2_Te_3_ requirement, but in none of them the vacancies are ordered. This explains why a phase transition from the amorphous phase to the hexagonal phase is prohibitively difficult, as one has to order the vacancies into periodic planes.^[^ ^28^ ^]^ In 2D ST, on the other hand, due to the spatial constraint the vacancies are retained at the interfaces between ST and substrate/superstrate even in amorphous samples. As such, it is no longer prohibitive to reorder them into the hexagonal phase.

**Figure 5:**
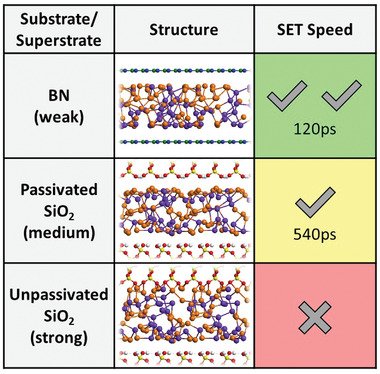
Effects of substrate and superstrate on the recrystallization (SET). (Top to bottom) Hexagonal BN, passivated SiO_2_, and unpassivated SiO_2_ are used as the substrate and/or superstrate, respectively. The figures show the amorphous structures and relative SET time (or speed). Color coding of Sb, Te, Si, O, and H is the same as in Figure 2, while green is for B and blue is for N.

Speaking of the substrate/superstrate, evidently phase transition in 2D PCM materials can be sensitive to surrounding environments. Here, we compare three different kinds of substrate/superstrate: hexagonal BN, H‐passivated SiO_2_ (001) surfaces, and unpassivated SiO_2_ (001) surfaces, in the order of increased interactions with the PCM material. We perform MD simulations for the first two cases in their entirety. To contrast the effects with and without the H passivation, we remove the passivation from the superstrate at *t* = 420 ps during a 600 K MD of H‐passivated SiO_2_ (001) simulation. **Figure** **5** tabulates the results, which shows that hexagonal BN, with the weakest interaction with the ST layer, finishes the SET (recrystallization) process in about 120 ps (see Figure S8 in the Supporting Information). H‐passivated SiO_2_ (001), with the second weakest interaction, finishes the SET process in about 540 ps. In contrast, when the H‐passivation in the superstrate is removed, no SET process can be observed throughout the MD of 480 ps (and the total MD time is 900 ps). As a matter of fact, in the latter case, the entire ST layer is lifted up and stuck on the superstrate (see Figure S9 in the Supporting Information). This result can be expected as the aforementioned p‐bonding segments, in particular, the 3‐ACs, are eliminated by the formation of heterogeneous chemical bonds with bare SiO_2_ (001) surface. We note that there is a possible stochastic nucleation process, so the comparison of time should be relative.

## 3 Conclusion

In summary, based on first‐principles molecular dynamics, we propose the physical mechanism of phase change in monolayer ST (0.8 nm), which is the thinnest PCM material to date. We show that, working at the 2D limit, both the crystallization speed and amorphous stability can benefit. It should be noted that recently experiments also start to shift their attention to scaling PCM materials toward such 2D limit, for example, the 3 nm thick antimony PCM material and optoelectronics based on it, ^[^ ^39^, ^47^ ^]^ as well as the 4 nm thick antimony PCM device.^[^ ^48^ ^]^ These materials also demonstrate potentials in future neuromorphic computing chips due to a considerably lowered resistance drift. Moreover, working with small volume PCM materials is an effective way to lower power consumption.^[^ ^18^, ^49^ ^]^ Regarding the experimental feasibility, different approaches, such as vapor deposition^[^ ^50^ ^]^ or liquid exfoliation,^[^ ^51^ ^]^ have been used to fabricate monolayer or few‐layer chalcogenide ST. Hence, by scaling the PCM materials to their 2D limit, our findings and physical understanding here will help to improve the performance of PCM technology in terms of the integrated density, speed, power consumption, and even artificial intelligence applications.

## 4 Experimental Section

The density functional theory (DFT) as implemented in the Vienna ab initio simulation package was employed.^[^ ^52^ ^]^ The electron‐ion interaction was described by the projector augmented wave pseudopotential.^[^ ^53^ ^]^ The electronic exchange–correlation interaction was described by the generalized gradient approximation with the Perdew–Burke–Ernzerhof functional.^[^ ^54^ ^]^ The calculations included the vdW interactions by using the Grimme's DFT‐D2 scheme.^[^ ^55^ ^]^ For the MD simulations, the NVT canonical ensemble was used, in which the Nosé‐thermostat is used to control the temperature.^[^ ^56^ ^]^ A 3 fs time step, an energy cutoff of 300 eV, and a single *k*‐point were used. The monolayer ST model contained thick enough vacuum layers to minimize interactions with its periodic images. The melt‐quench method was used to obtain amorphous 2D ST. To mimic a device, the monolayer ST was sandwiched between substrate and superstrate of the same material.

According to the strength of the interaction, three kinds of substrates/superstrates were used, i.e., unpassivated SiO_2_ (001) surfaces, passivated SiO_2_ (001) surfaces,^[^ ^34^ ^]^ and 2D hexagonal BNs. To get a stable 2D amorphous form, crystalline ST was melted at 3000 K for 9 ps and then cooled down to 1200 K. Next, the liquid was equilibrated at this temperature for 18 ps, quenched to 300 K, and then maintained for another 15 ps. To reduce massive load of calculations, the substrate/superstrate was fixed during the amorphization. To mimic the SET (recrystallization) process, the MD was run for a prolonged time (300–900 ps) at 600 K. For structural analyses, a 1.2× (sum of covalent radii) was used as the bond length cutoff at 300 K and 1.3× (sum of covalent radii) at 600 K (recrystallization). A larger cutoff was needed for 600 K due to the stronger fluctuation of atoms. More details about the modeling are given in Section 1 in the Supporting Information.

## Conflict of Interest

The authors declare no conflict of interest.

## Supporting information



Supporting Information

## Data Availability

The data that support the findings of this study are available from the corresponding author upon reasonable request.
