# Supplementary material for: Phase‐Change‐Memory Process at the Limit: A Proposal for Utilizing Monolayer Sb2Te3
Source: Adv Sci (Weinh). 2021 May 14;8(13):2004185. doi: 10.1002/advs.202004185 (PMC8261487; doi:10.1002/advs.202004185)
Supplement: Supplementary file 1 — Supporting Information [file ADVS-8-2004185-s001.pdf]

© 2021 Wiley-VCH GmbH

ADVANCED SCIENCE
Open Access

Supporting Information

for *Adv. Sci.,* DOI: 10.1002/advs.202004185

# Phase-Change-Memory Process at the Limit: A Proposal for Utilizing Monolayer $Sb_2Te_3$

*Xue-Peng Wang,Xian-Bin Li*, Nian-Ke Chen*,Bin Chen, Feng Raoand Shengbai Zhang**

© Copyright 2020. WILEY-VCH GmbH.

Supporting Information

**Phase-Change-Memory Process at the Limit: A Proposal for Utilizing Monolayer $Sb_2Te_3$**

*Xue-Peng Wang,Xian-Bin Li*, Nian-Ke Chen*,Bin Chen, Feng Raoand Shengbai Zhang**

## 1. Modeling details

**Table S1**Lattice parameters of monolayer $Sb_2Te_3$ sandwiched by passivated $SiO_2$ (001) and hexagonal BN, respectively.

| | *a, b* | *c* | *α, β* | *γ* | Distance |
|---|---|---|---|---|---|
| $Sb_2Te_3$ - passivated $SiO_2$ | 14.9 Å | 25.021 Å | 90° | 120° | 3.20 Å |
| $Sb_2Te_3$ - hexagonal BN | 14.9 Å | 25.021 Å | 90° | 120° | 3.34 Å |

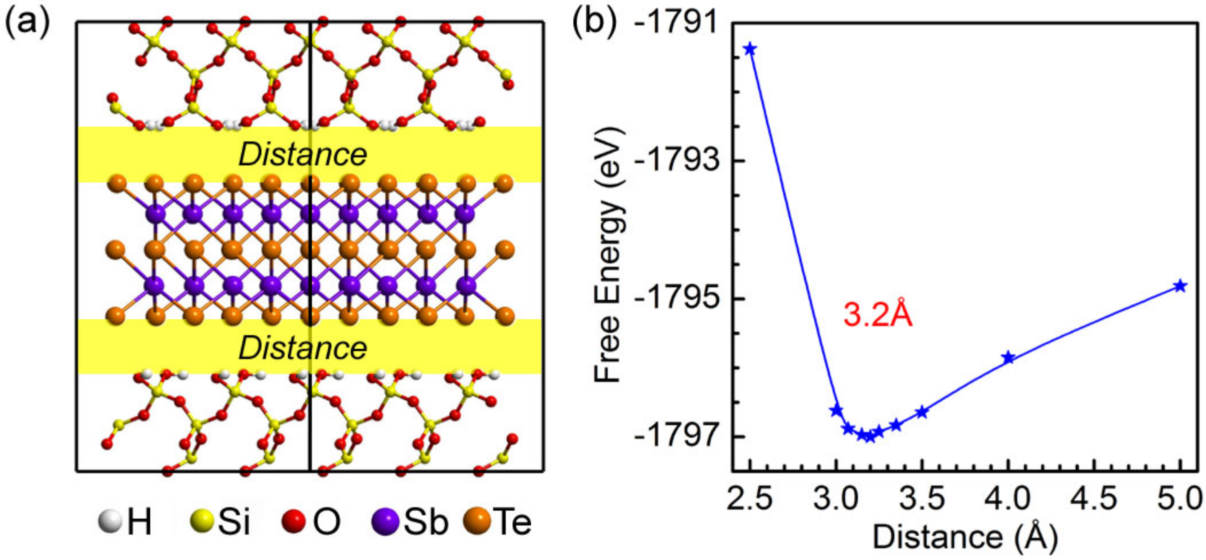


**Figure S1.** (a) Structure of monolayer $Sb_2Te_3$ sandwiched by passivated $SiO_2$ (001). Atom color coding: white for H, yellow for Si, red for O, purple for Sb, and orange for Te. (b) Free energy versus the distance between monolayer $Sb_2Te_3$ and $SiO_2$ [yellow areas in (a)].

Table S1 shows the lattice parameters of the monolayer $Sb_2Te_3$ (ST) sandwiched by passivated $SiO_2$ (001) (an 18-layer hydroxylated α-$SiO_2$ slab given by Ref. [1]) and hexagonal BN, respectively. The distance between monolayer $Sb_2Te_3$ and $SiO_2$ or BN is determined by energy minimization, as shown in FigureS1(b).

## 2. Atomic coordination number distribution in3D and 2D amorphous ST

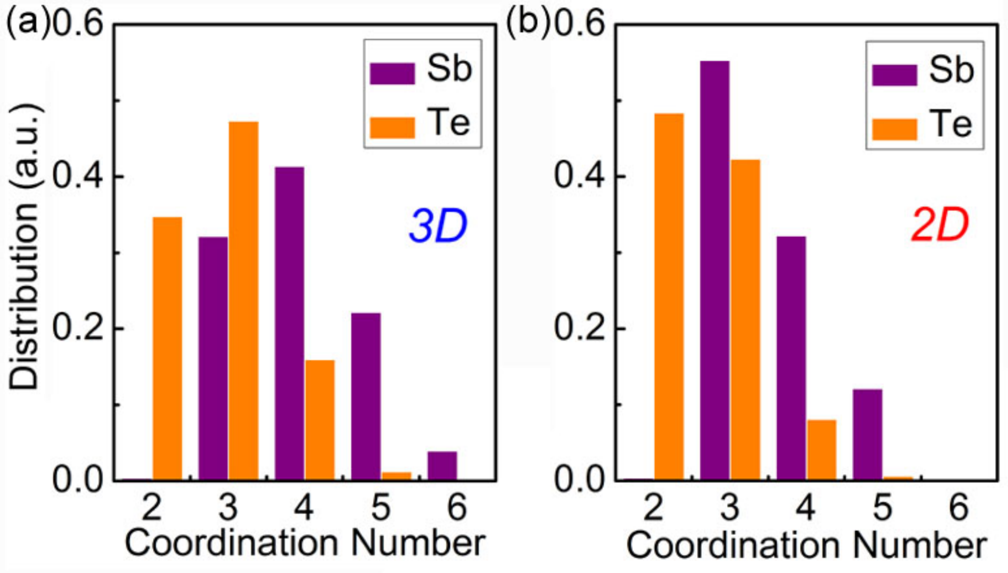


**Figure S2.** Coordination number (CN) distributions of Sb and Te in (a) 3D and (b) 2D amorphous ST.

As shown in FigureS2(a) and (b), for the 3D amorphous ST, 4fold-coordinated Sb and 3fold-coordinated Te dominate. In contrast, for the 2D amorphous ST, 3fold-coordinated Sb and 2fold-coordinated Te dominate. Higher CNs such as 5fold or 6fold are considerably less in 2D amorphous ST. There are two reasons that lead to fewer atoms with high CN in 2D amorphous ST: on the one hand, 2D ST has more atoms on the surface which have fewer neighbors. On the other hand, the ELF distribution reveals electrons are more localized and in form of lone-pairs in 2D amourphous ST. The lone-pairs electrons also reduce the CN.

## 3. The distributed volume of electrons at a given ELF value

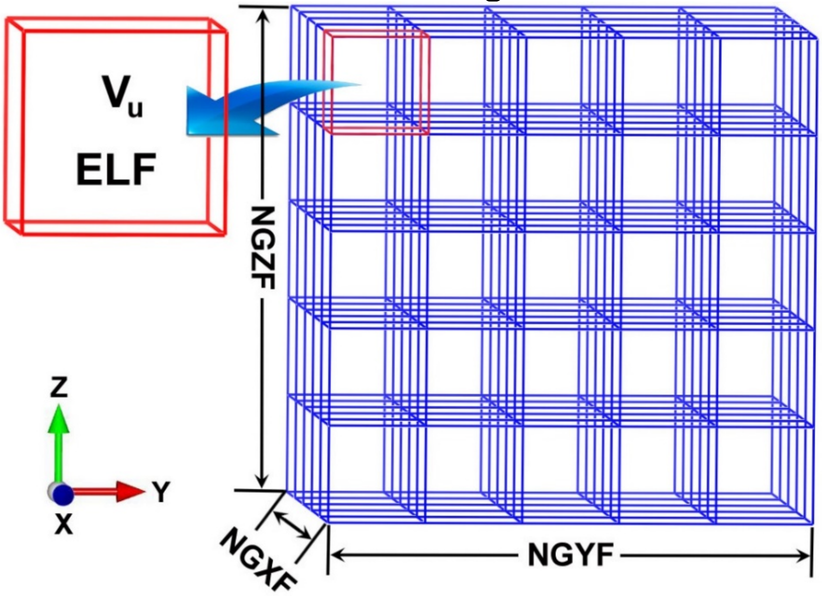


**Figure S3.** Schematic diagram of calculating the distributed volume of electrons at a given ELF value from the ELFCAR of the VASP code.

In the VASP calculation, the calculation model is equally divided into many grid points. The electron localization function (ELF)[2] distribution is written in the output ELFCAR file which is defined on those grid points. As shown in Figure S3, each grid has an ELF value. The volume corresponding to each grid can be approximated by,

$$V_u = \frac{V_{cell}}{NGXF \times NGYF \times NGZF}$$

where $V_{cell}$ is the volume of the calculation cell, *NGXF*, *NGYF*, *NGZF* are the numbers of grid points along X, Y and Z directions, respectively. So, the amount of total grid points is equal to *NGXF*×*NGYF*×*NGZF*. Therefore, we can select the grids with a certain ELF value, and thensum up the total volume of these grids. In this way, the distributed volume of electrons at any range of ELF value [in Figure 2(d) of the main text] can be calculated.

## 4. Lone-pairs in amorphous ST

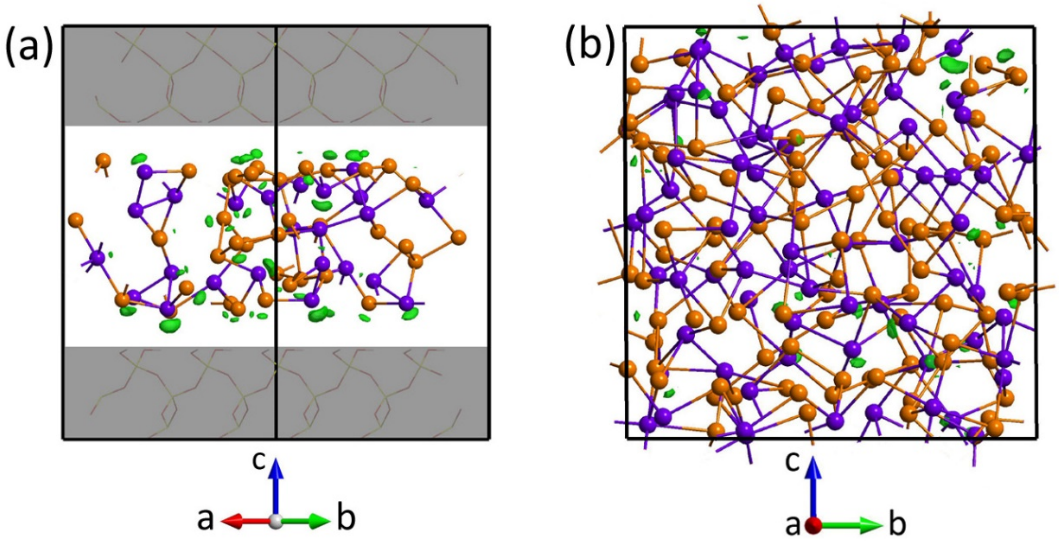


**Fig S4.** Distribution of highly-localized electrons in (a) 2D and (b) 3D amorphous ST, as reflected by the green isosurface at an ELF value = 0.93. Color coding of the atoms is the same as in Figure S1.

FiguresS4(a) and (b) display an example of highly-localized electrons in 2D and 3D amorphous ST with ELF ≥ 0.93. These electrons have cap-like shapes residing in local regions lacking of chemical bonds. These features are characteristic of the lone-pairs.[3,4]

## 5. Stability of 2D amorphous ST

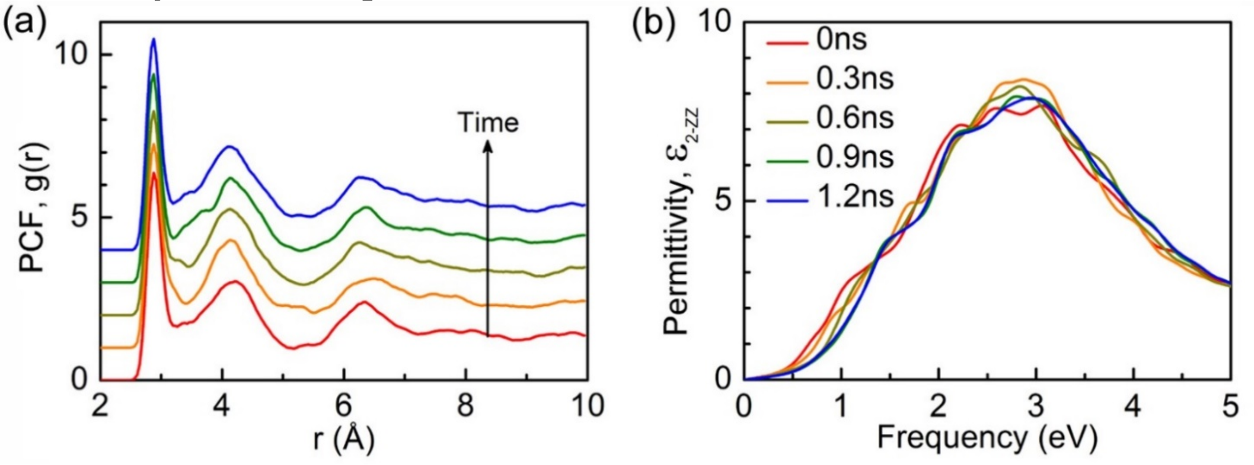


**FigureS5.** Time evolution of structural and optoelectronic properties of 2D amorphous ST during 300K annealing. (a) Pair correlation functions, PCFs and (b) the imaginary part of the permittivity, $\varepsilon_{2\text{-}ZZ}$ at 0 ns (red), 0.3 ns (orange), 0.6 ns (dark yellow), 0.9 ns (green), and 1.2 ns (blue).

In order to examine stability, 2D amorphous ST is annealed at 300K for 1.2ns by first-principles molecular dynamics (MD)simulations. As shown in FigureS5(a), the pair correlation functions (PCFs), especially their peak positions, are almost unchanged during the annealing, which means the 2D amorphous ST holds a good structural stability. Figure S5(b) shows the imaginary part of the permittivity along z direction ($\varepsilon_{2\text{-}ZZ}$), which is also almost unchanged. These suggest that the 2D amorphous ST is stable at room temperature.

## 6. Evolution of energy and some structural order parameters during crystallization process

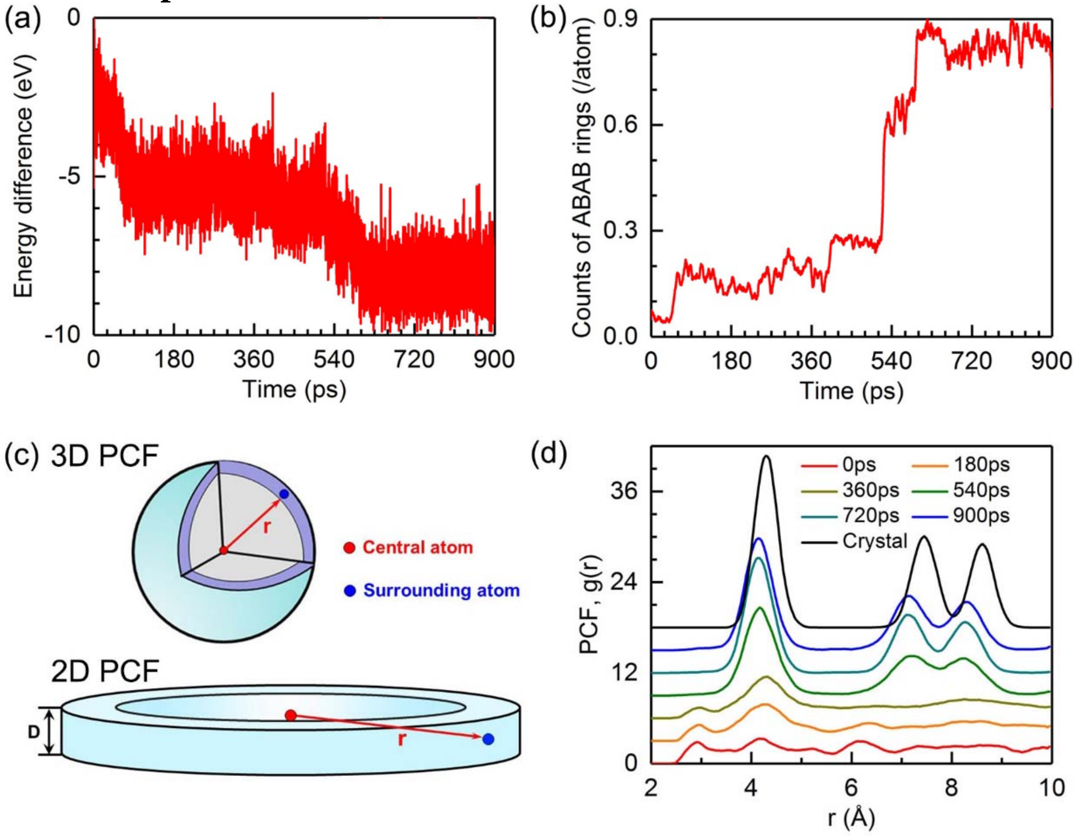


**Figure S6.** Evolution of (a) energy and (b) counts of 4-fold ABAB atom rings during crystallization process of 2D ST. (c) Schematic diagram of 3D and 2D PCF. D stands for the height of the hollow cylinder shell in 2D PCF calculation (d) 2D PCF at different time of recrystallization. The black curve is the 2D PCF of a perfect monolayer ST crystal.

Figure S6(a) displays the energy evolution during crystallization process. The energy shows a significant drop at about 540 ps which implies crystallization occurs. This is consistent with the results of z-coordinate distribution. We also investigate crystallization process with other order parameters, such as 4-fold ABAB atom rings and 2D PCF. The ABAB rings are also a sign of crystalline phase. As shown in Figure S6(b), lots of ABAB rings form at about 540ps. Apart from the significant increase at 540ps, the amount of ABAB rings also show a slight increase at about 420ps that can be attributed to the formation of reoriented 3-atom-chains template. In addition, we also propose a 2D PCF method to describe the structural order in the x-y plane. As displayed in Figure S6(c), a traditional 3D PCF describes the density of surrounding atoms in a thin spherical shell with a thickness of dr change with the distance between a surrounding atom and a central atom (*i.e.*r). A 3D PCF $[g^{3D}(r)]$ can be expressed as

$$g^{3D}(r)=\frac{\rho_{shell}}{\rho}\approx\frac{N_r/(4\pi r^2\times dr)}{\rho}.$$

Where $\rho_{shell}$ and $\rho$ stands for the density of surrounding atoms in the spherical shell and that in the whole system, respectively. $N_r$ is the amount of surrounding atoms in the spherical shell with a radius of r. For a 2D PCF shown in the lower case of Figure S6(c), we employ a hollow cylinder shell with thickness of dr to replace the spherical shell in 3D PCF. To mainly count the surrounding atoms in the x-y plane, the height of the hollow cylinder shell (D=0.46375Å) is selected as 1/16 of the thickness of 2D crystalline ST. Therefore, the 2D PCF for a central atoms can be written as

$$g^{2D}(r)=\frac{\rho_{shell}}{\rho}\approx\frac{N_r/(2\pi r\times dr\times D)}{\rho}.$$

Note that, the position of the first peak of 2D PCF depends on the structure feature of 2D material. For example, the position of the first peak of 2D PCF is determined by the second closest atoms in a 2D ST crystalline phase because the first closet bonding connection is not in-plane. Despite this, the 2D PCF could clearly reflect the in-plane order of a 2D system. Figure S6(d)displays the 2D PCF during crystallization. There are no peaks of 2D PCF that suggest long range order until 540ps. And the peak positions of 2D PCF after 540ps matches well with those for a perfect crystalline phase. Therefore, a crystalline phase with long range order in both in-plane (2D PCF) and out-of-plane (z-coordinate) directions has established after 540ps.

## 7. Orientation of atom chains during crystallization of 2D amorphous ST

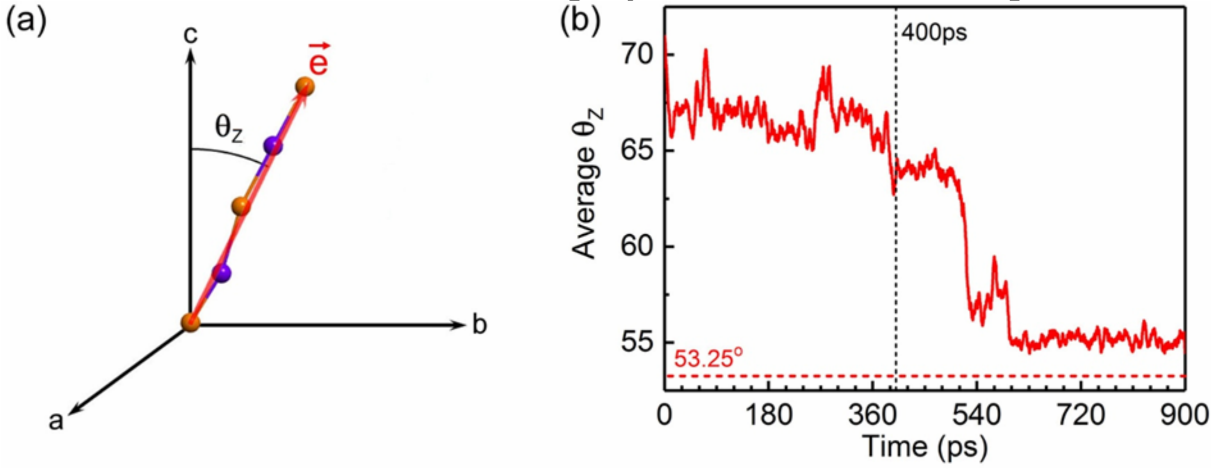


**FigureS7.** (a) A diagram showing the direction vector ($\vec{e}$) of an atom chain and its angle ($\theta_Z$) with the z-axis. (b) Time evolution of the average $\theta_Z$ during a 600K recrystallization of 2D amorphous ST. Red dash line [$\theta_Z$(5-AC) = 53.25°] is the value for monolayer crystalline ST.

For the atom chain (AC) analysis, the AC is defined as such an arrangement of atoms where their bond lengths are less than 3.6 Å and bond angles deviate less than 20° from 180°.[5]As shown in Figure S7(a), the direction vector ($\vec{e}$) of the AC is determined by the acute angle $\theta_Z$between $\vec{e}$ and the positive direction of the z-axis. For a perfect monolayer crystalline $Sb_2Te_3$, which contains exclusively 5-atom chains (5-AC), $\theta_Z$would be 53.25°. Figure S7(b) shows the evolution of the average $\theta_Z$ during the recrystallization of 2D amorphous ST. At the beginning, the average $\theta_Z$ is much larger than the crystalline angle of 53.25° due to disorder. At 540 ps, $\theta_Z$drops significantly towards the 53.25°, signaling the onset of a crystallization. In addition, we also find a decrease of $\theta_Z$at an earlier time of 400 ps (noted by a vertical black dash-line), which is a sign that noticeable amount of 3-atom chains (3ACs) have reoriented towards ordering.

## 8.Effects of substrate/superstrate on crystallization of 2D amorphous ST

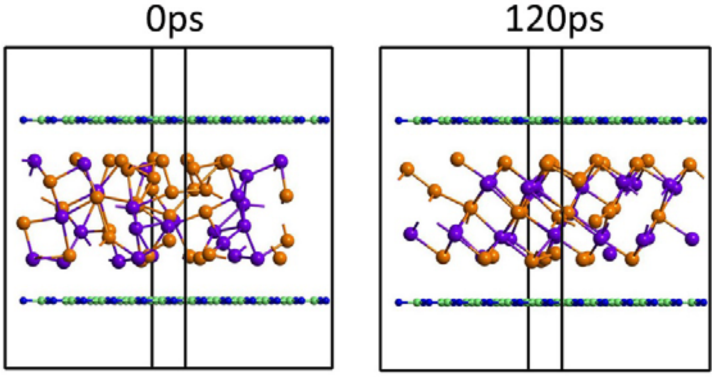


**FigureS8.**600K recrystallization MD of 2D amorphous ST sandwiched between hexagonal Boron Nitride(BN). Snapshots of the structures at 0 ps (amorphous phase) and 120 ps (crystalline phase) are shown.

With a hexagonal BN sandwiching, quintuple-layered structure can clearly be seen after about 120 ps.

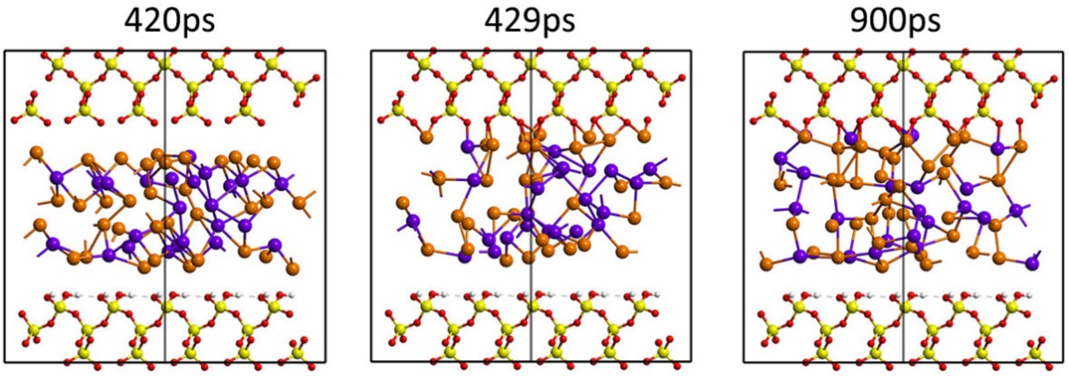


**FigureS9.** 600K recrystallization MD of 2D amorphous ST sandwiched between hydrogenated $SiO_2$, but the passivating H atoms on the superstrate are removed at t = 420 ps. The MD is up to 900 ps. Snapshots of the structures at 420 ps, 429 ps, and 900 ps are shown.

After removing H passivation from the superstrate at 420 ps, the ST layer is quickly uplifted as a whole, due to the formation of strong chemical bonds with unpassivated O, as shown in the structure at 429 ps. The 2D ST also remains disordered in the entire 900 ps annealing.

## References

[1] T. P. M. Goumans, A. Wander, W. A. Brown, C. R. A. Catlow, *Phys. Chem. Chem. Phys*. **2007**, *9*, 2146.
[2] A. Savin, O. Jepsen, J. Flad, H. G. von Schnering, *Angew. Chem. Int. Ed. Engl.***1992**, *31*, 187.
[3] X. P. Wang, X. B. Li, N. K. Chen, Q. D. Chen, X. D. Han, S. B. Zhang, H. B. Sun,*Acta Mater.***2017**, *136*, 242.
[4] X. P. Wang, Y. T. Liu, Y. J. Chen, N. K. Chen, X. B. Li, *J. Phys. D: Appl. Phys.***2020**, *53*, 114002.
[5] N. K. Chen, X. B. Li, X. P. Wang, W. Q. Tian, S. B. Zhang, H. B. Sun, *Acta Mater.***2018**, *143*, 102.
